# Deep learning estimation of proton stopping power with photon-counting computed tomography: a virtual study

**DOI:** 10.1117/1.JMI.11.S1.S12809

**Published:** 2024-11-20

**Authors:** Karin Larsson, Dennis Hein, Ruihan Huang, Daniel Collin, Andrea Scotti, Erik Fredenberg, Jonas Andersson, Mats Persson

**Affiliations:** a KTH Royal Institute of Technology, Department of Physics, Stockholm, Sweden; b Karolinska University Hospital, MedTechLabs, BioClinicum, Solna, Sweden; c GE HealthCare, Stockholm, Sweden; d Umeå University, Department of Diagnostics and Intervention, Radiation Physics, Umeå, Sweden

**Keywords:** photon-counting computed tomography, proton therapy, proton stopping power, deep learning

## Abstract

**Purpose:**

Proton radiation therapy may achieve precise dose delivery to the tumor while sparing non-cancerous surrounding tissue, owing to the distinct Bragg peaks of protons. Aligning the high-dose region with the tumor requires accurate estimates of the proton stopping power ratio (SPR) of patient tissues, commonly derived from computed tomography (CT) image data. Photon-counting detectors for CT have demonstrated advantages over their energy-integrating counterparts, such as improved quantitative imaging, higher spatial resolution, and filtering of electronic noise. We assessed the potential of photon-counting computed tomography (PCCT) for improving SPR estimation by training a deep neural network on a domain transform from PCCT images to SPR maps.

**Approach:**

The XCAT phantom was used to simulate PCCT images of the head with CatSim, as well as to compute corresponding ground truth SPR maps. The tube current was set to 260 mA, tube voltage to 120 kV, and number of view angles to 4000. The CT images and SPR maps were used as input and labels for training a U-Net.

**Results:**

Prediction of SPR with the network yielded average root mean square errors (RMSE) of 0.26% to 0.41%, which was an improvement on the RMSE for methods based on physical modeling developed for single-energy CT at 0.40% to 1.30% and dual-energy CT at 0.41% to 3.00%, performed on the simulated PCCT data.

**Conclusions:**

These early results show promise for using a combination of PCCT and deep learning for estimating SPR, which in extension demonstrates potential for reducing the beam range uncertainty in proton therapy.

## 1 Introduction

It is estimated that around half of cancer patients receive radiation therapy as part of their treatment plan, making it one of our most indispensable cancer treatment modalities.^1^ Conventional radiotherapy employs high-energy photons for administration of radiation dose and accounts for around 99% of all radiation treatments.^2^ Although photon radiotherapy has seen great progress in tumor control and sparing of normal tissue, an impediment to the latter objective is the continuous deposition of dose along the beam trajectory through the patient.^2^ This may pose a clinical challenge when considering organs at risk and selecting optimal beam angles.

By contrast, protons could allow for a more precise dose delivery to the tumor. Protons exhibit sharp Bragg peaks when traversing matter before coming to a stop, which concentrates the radiation dose to the tumor while sparing normal tissue distal to the dose fall-off. A prerequisite for such a selective dose deposition is a minimal proton beam range uncertainty, for which the estimation of the stopping power ratio (SPR) relative to the water of tissues is a major factor.

In clinical practice, the SPR values of patient tissues are estimated from Hounsfield units (HU) in computed tomography (CT) images. Due to the inherently different interaction mechanisms of photons and protons, this conversion is a significant source of uncertainty. To ensure coverage of the clinical target volume, it is common practice to take beam range uncertainty into account by applying a relative margin (ca 2.5% to 3.5%), an absolute margin (1 to 7 mm), or a combination of the two.^3^^,^^4^ Reducing the beam range uncertainty would allow for smaller margins, leading to decreased radiation burden to normal tissue and associated side effects.^2^^,^^5^ To fully exploit the potential precision of proton therapy, it is therefore of interest to develop accurate and robust methods of conversion from HU to SPR.

To date, the conventional method for conversion in the clinic has been single-energy CT (SECT) stoichiometric calibration, as described in Ref. 6. A parameter model connecting material properties with HU is solved by scanning tissue substitutes, after which data pairs of HU and SPR are calculated based on the solved model and listed compositions for human reference tissues. A piecewise linear calibration curve is obtained through linear regression of each category of tissues, which then serves as a HU lookup table (HLUT) used to convert HU to SPR values. The approach assumes a bijective relationship between HU and SPR, which both have separate dependencies on tissue properties relating to the elemental composition.^7^ Because these dependencies cannot be fully elucidated by a one-to-one relation, it renders the HLUT method sensitive to variations between the reference tissues and real patient tissues, with reported combined range uncertainties between 2.4% (+1.2  mm) and 3.4%.^4^^,^^8^

Spectral imaging techniques, i.e., dual-energy CT (DECT) and photon-counting CT (PCCT), enable a more rigorous tissue characterization, which may address these concerns. Experimental validation of DECT-based methods typically reports root mean square errors (RMSEs) for SPR estimates of 0.7% to 1.5%^9^^–^^11^ or an accuracy of about 0.6%.^12^ Moreover, one study showed that DECT-based methods reduced the error for the beam range in water by up to 0.4 p.p. compared with SECT.^7^ On the other hand, the same study found that DECT was outperformed by SECT for higher noise levels. Consequently, DECT has demonstrated potential for improving SPR estimation, provided that the image data is sufficiently free from noise and artifacts.^7^^,^^13^^,^^14^

While energy-integrating detectors (EIDs) are the convention in CT, there has been advancement of photon-counting detectors (PCDs) in recent years. Where EIDs rely on indirect conversion that integrates the energy of incident photons, PCDs utilize semi-conductors and integrated circuits to count and measure the energy of each photon.^15^ The resulting detection technique has several advantages, not least for spectral imaging. By measuring the energy of each incoming photon, PCDs make use of the polyenergetic nature of the X-ray beam and therefore do not require changes to the X-ray source or imaging protocols to perform spectral imaging. Problems associated with DECT, such as cross-scattering of photons in a dual-source system, or low spectral separation with fast kV-switching sources and dual-layer detectors,^16^ are thus not introduced in PCCT.

Spectral imaging techniques make it possible to infer information about the material composition, which may be of particular interest for SPR estimation. Based on the material decomposition, it is then possible to create virtual mono-energetic images (VMIs) that imitate the result a true mono-energetic photon beam would produce, which effectively reduces beam-hardening artifacts. The material decomposition and VMI accuracy depend largely on the energy resolution, where PCCT is expected to have an advantage due to the improved spectral separation and not being limited to two independent energy measurements.^15^^,^^17^ Furthermore, the electronic noise can be rejected by placing the lowest threshold for detection just above the electronic noise level, whereas superior spatial resolution can be achieved because reflective septa between pixels are not required, as described in Ref. 17. Recent work has also reported a high robustness to noise for PCCT-based methods for SPR estimation.^18^^,^^19^

The objective of this work was to provide a proof of concept for a combination of PCCT and supervised deep learning to estimate SPR from CT image data and compare the performance to that of existing methods. Deep learning has seen great advancement in the realm of CT reconstruction and correction of artifacts, owing to neural networks’ unparalleled ability to decipher subtle relations from large data sets. Through high-quality CT image data with a simulated PCD, a neural network can be provided with implicit information about the elemental composition of the patient tissues. Using simulated data and digital phantoms, we calculate ground truth SPR maps that can be provided as labels. Viewing the CT image data as a representation of the tissue composition, the idea is to let a neural network learn the domain transform from PCCT image data to SPR values. Because the network may simultaneously learn to recognize and correct for imaging uncertainty, such as noise, it may also allow for a more robust method of conversion compared with previous DECT methods that are more sensitive to image noise. A preliminary, more limited version of this work was published in the proceedings of SPIE.^20^

## 2 Methods

### 2.1 XCAT Phantom Definition

Voxelized phantoms were generated using the XCAT phantom program developed by Segars et al. at Duke University.^21^ Because many cancer indications relevant for treatment with proton therapy involve tumors in, or in close proximity to, the brain, spinal cord, or other constituents of the central nervous system, the head was chosen as the focus region in this work. The phantom anatomy used defined the skull and brain region, with skull and skin thicknesses set to 6.5 and 2.5 mm, respectively.^22^

To model anatomical variation, six different XCAT phantoms were included in the data set (three male and three female). One male (“vmale50”) and one female phantom (“vfemale50”) were of standard 50th percentile geometries while the remaining phantoms (“male_pt77,” “male_pt148,” “female_pt86,” and “female_pt147”) introduced further variation. The male phantoms had BMIs of 22.71, 24.38, and $32.18\;\mathrm{kg}/m^{2}$, and the female phantoms 18.21, 24.06, and $38.81\;\mathrm{kg}/m^{2}$. However, because this work focused on the head, neither BMI or gender was expected to contribute significantly to the variation, and a more meaningful measure is the range of head sizes included. The maximal geometrical diameters of the phantoms were 203, 208, 209, 215, 223, and 224 mm, and maximal water equivalent thicknesses were 187, 193, 193, 200, 204, and 206 mm.^23^ The anatomical variation of the XCAT phantoms is generated from the PeopleSize program, based on anthropometric dimensions of individuals from several countries.^24^ From each phantom, 281 slices were selected, yielding a data set of 1686 examples.

The XCAT program includes the functionality of “activity” phantom generation, originally intended for nuclear medicine simulations. The output is a three-dimensional matrix where voxels of a delineated body structure are assigned the same number. This feature was used to assign each voxel a known tissue material, with compositions in accordance with Appendix A from ICRU Report 46.^25^

To introduce substructure in the brain, the brain structures were classified as white or gray matter. If the structure contained a mix, it was assigned the whole brain material from Ref. 25. The white and gray matter materials were elementally defined according to densities and compositions listed in Ref. 26. The XCAT phantom included a background structure, comprising the space between the brain and the skull, as well as between the skull and the skin. The background was set to adipose, in accordance with pre-set values for the attenuation phantom generation by the XCAT program.

The phantom pixel width and slice width were set to 0.25 and 0.416 mm, respectively. This choice was motivated by the desire to keep the phantom resolution similar to or higher than the size of the reconstructed pixels. In addition, using a slice thickness equal to that of the reconstructed images facilitated the pairing of corresponding CT and ground truth SPR slices.

### 2.2 Ground Truth SPR Maps

Based on the known material composition in each voxel, the XCAT phantom was converted to ground truth SPR maps. The relative electron density and mean ionization energy of the materials were calculated as

$$\rho_{e}=\frac{\rho N_{A}\underset{i}{\sum }\omega_{i}\frac{Z_{i}}{A_{i}}}{\rho_{e,w}},$$ (1)

$$I=\exp[(\underset{i}{\sum }\omega_{i}\frac{Z_{i}}{A_{i}}\;\ln(I_{i}))/(\underset{i}{\sum }\omega_{i}\frac{Z_{i}}{A_{i}})],$$ (2)

where $\rho_{e}$ denotes relative electron density, ρ the density of the material, $N_{A}$ Avogadro’s constant, $\omega_{i}$ the elemental weights by mass, $Z_{i}$ the atomic number of element i, $A_{i}$ the atomic weight of element i, $\rho_{e,w}$ the relative electron density of water, and I the mean ionization energy. Elemental ionization energies were primarily taken from Ref. 27, and for elements not listed, values were taken from Ref. 28. These entities were then used to compute the ground truth SPR using the Bethe-Bloch formula,

$$\mathrm{SPR}=\rho_{e}[\left(\ln\;\frac{2m_{e}c^{2}\beta^{2}}{(1-\beta^{2})I}-\beta^{2}\right)/(\ln\;\frac{2m_{e}c^{2}\beta^{2}}{(1-\beta^{2})I_{w}}-\beta^{2})],$$ (3)

where $m_{e}$ denotes the mass of the electron and β the ratio of the proton speed relative to the speed of light. β was set to 0.461376419, corresponding to a proton kinetic energy of 100 MeV (non-relativistic calculation).

**Fig. 1:**
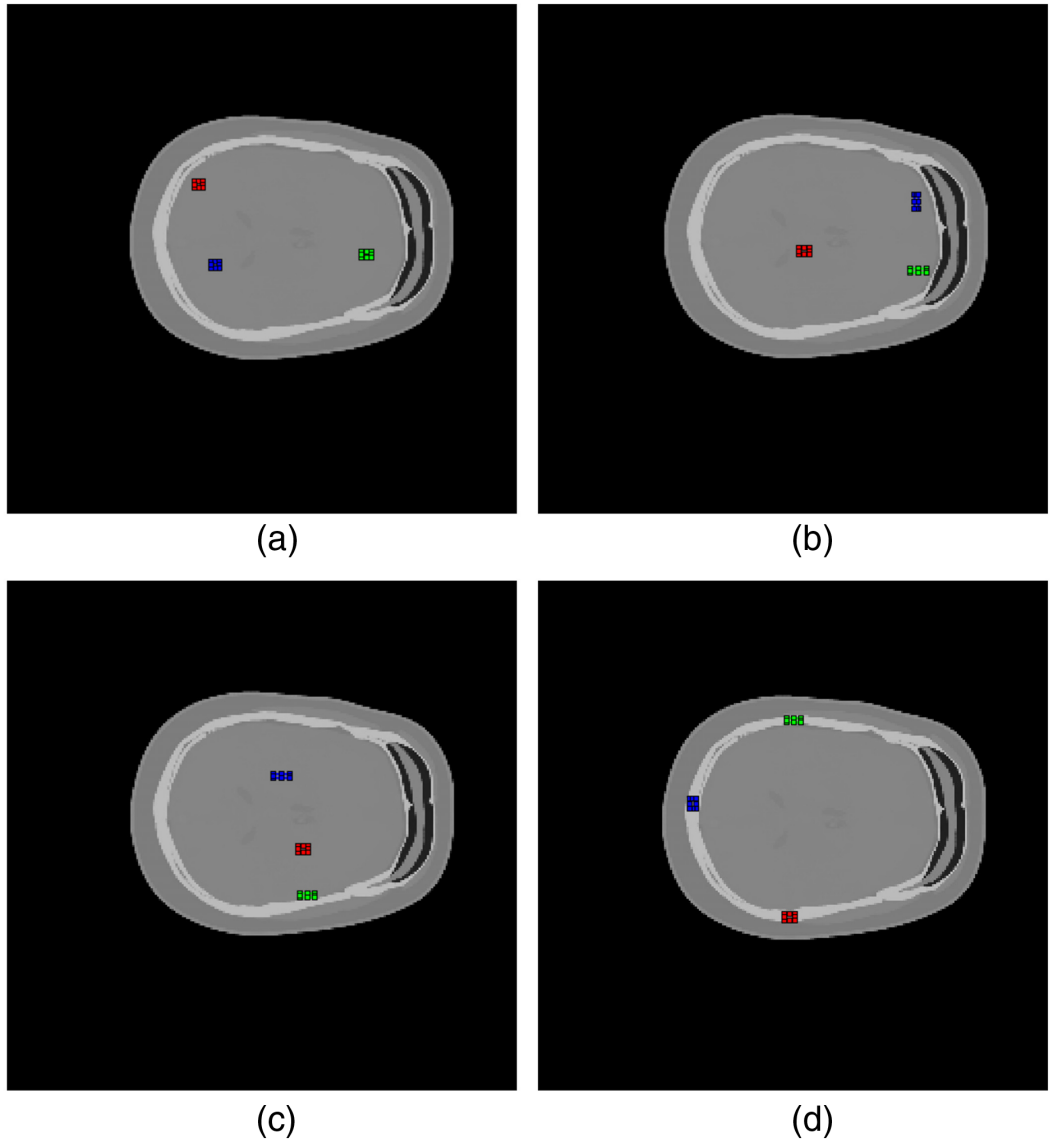
ROIs of the (a) white matter, (b) gray matter, (c) whole brain, and (d) skull materials. For all materials, ROIs 1, 2, and 3 are drawn as red, blue, and green, respectively.

Three soft tissue materials (white matter, gray matter, and whole brain) and the skull were selected for evaluation of SPR accuracy. Three regions of interest (ROI) for each material were selected in one slice. RMSE, relative error, and relative standard deviation were calculated as percentages for each ROI, with formulas given by

$$\mathrm{RMSE}=\sqrt{\frac{1}{N}\sum ({\mathrm{SPR}}_{\text{truth}}-{\mathrm{SPR}}_{\mathrm{est}}{)}^{2}}*100\%,\;\text{Relative error}=\frac{1}{{\mathrm{SPR}}_{\text{truth}}}|{\mathrm{SPR}}_{\text{truth}}-{\overset{¯}{\mathrm{SPR}}}_{\mathrm{est}}|*100\%,\;\text{Relative standard deviation}=\frac{1}{{\mathrm{SPR}}_{\text{truth}}}\sqrt{\frac{\sum ({\mathrm{SPR}}_{\mathrm{est}}-{\overset{¯}{\mathrm{SPR}}}_{\mathrm{est}}{)}^{2}}{N-1}}*100\%,$$ (4)

where ${\bar{\mathrm{SPR}}}_{\mathrm{est}}$ denotes the mean of estimated SPR values in the ROI and N the number of pixels in the ROI. The ROIs and the number of pixels of each are shown in Fig. 1 and Table 1, respectively.

**Table 1:** Number of pixels in the ROIs.

Material	ROI 1	ROI 2	ROI 3	Total
White matter	20	20	24	64
Gray matter	24	24	27	75
Brain (whole)	24	27	24	75
Skull	24	24	24	72

### 2.3 Photon-Counting CT Simulations

Photon-counting CT imaging of the XCAT phantom was performed in CatSim^29^ using a proprietary simulation model of a deep-silicon-based photon-counting CT prototype system developed by GE HealthCare.^30^ An open-source version of CatSim, with more limited functionality, is available online.^31^

The simulations were defined by parameters corresponding to a realistic CT protocol for a head scan. The tube current, rotation time, tube voltage, and number of view angles were set to 260 mA, 1 s, 120 kV, and 4000, respectively. We simulated an axial scan with 5 mm coverage, using an approximation of the GE LightSpeed large bowtie filter (“VCTlarge.txt”). For each view and detector pixel, a single projection line was simulated.

Physical phenomena that are known to impact image quality can be included in the simulations, such as electronic noise (manifested as Poisson-distributed counts in the lowest energy bin), quantum noise, pileup (photons arriving at the detector during the dead time following the registration of a previous photon, which consequently go unregistered and/or distort the detected energy spectrum), and crosstalk (charge sharing and reabsorption of Compton scattered photons). The simulations in this study included electronic and quantum noise, but excluded the effects of crosstalk and pileup, to avoid an excessively long simulation runtime. In other words, we assume that the system has a perfect correction for artifacts due to crosstalk and pileup.

Simulating voxelized phantoms in CatSim requires files that describe the phantom in terms of resolution and size while referring to material density matrices that provide the fractions of a given material at each image point. A MATLAB script was used to convert the raw XCAT output to the format required by CatSim. To reduce memory and compute time requirements, a material decomposition into polyethylene (PE) and polyvinyl chloride (PVC) was performed to reduce the number of density matrices to two. The choice of basis materials was motivated by PE and PVC being commonly used calibration materials since they can be linearly combined to approximate human tissues with positive coefficients.^32^

The linear attenuation coefficient at any point in the body can be approximated by a linear combination of basis functions, which can be expressed as

$$\mu (E)=\sum_{j=1}^{N_{m}}a_{j}f_{j}(E),$$ (5)

where $\mu ,N_{m},a_{j}$ and $f_{j}(E)$ denote the linear attenuation coefficient, the number of basis functions, basis coefficients, and basis functions, respectively.

The material decomposition into PE and PVC was performed by first calculating their respective linear attenuation coefficients at 40 and 70 keV using the “GetMu” function available in the XCIST repository on GitHub, as well as corresponding coefficients for the materials in the XCAT phantom. For each point, an equation system

$$a_{1}\mu_{\mathrm{PE}}(40\;\mathrm{keV})+a_{2}\mu_{\mathrm{PVC}}(40\;\mathrm{keV})=\mu (40\;\mathrm{keV})\;a_{1}\mu_{\mathrm{PE}}(70\;\mathrm{keV})+a_{2}\mu_{\mathrm{PVC}}(70\;\mathrm{keV})=\mu (70\;\mathrm{keV}),$$ (6)

was solved in MATLAB using the built-in “linsolve” function, yielding two basis coefficient maps with $a_{1}$ and $a_{2}$ denoting the fractions of respective basis material in each point, $\mu_{\mathrm{PE}}$ and $\mu_{\mathrm{PVC}}$ denoting the linear attenuation coefficients for PE and PVC, and μ the total linear attenuation coefficient. The coefficient maps were then used as density maps for the voxelized phantom in CatSim.

The forward projection was simulated in CatSim, and the image reconstruction was performed using a proprietary reconstruction prototype software for photon-counting CT. Based on registered photons in each energy bin, a material decomposition into PE and PVC was computed using a projection-space maximum-likelihood-based material decomposition algorithm, and basis images were reconstructed through filtered back-projection with a standard kernel. The outputs of the reconstruction software were water and iodine basis images, i.e., by default the software performed a change of basis from PE and PVC to water and iodine. This step was reversed using the inverse basis transformation matrix, and we used the initial PE and PVC basis images to compute the final reconstructed VMIs. The reconstruction field of view was set to 350 mm and the reconstructed slice thickness was 0.4167 mm. We did not apply any noise reduction algorithm to the image data.

Inspired by the work of Näsmark and Andersson, as described in Ref. 33, VMIs of 40 and 70 keV were used as input to the network. The basis images were therefore converted to 40 and 70 keV VMIs through linear combination, consistent with Eq. (6).

### 2.4 Neural Network Model

The network used in this project was a U-Net inspired by a network architecture employed as a component of denoising diffusion probabilistic models (DDPMs), as described by Song et al. in Ref. 34, who based their architecture on Ho et al. in Ref. 35. While using this network as a starting point, adaptations were made to employ the architecture for image-to-image translation that could be trained with the L1, MSE, and VGG16 loss functions. A necessary change was to remove the time embeddings used for the score-matching in diffusion models. The network had one BigGAN residual block per resolution and did not employ the anti-aliasing based on finite impulse response for up- and down-sampling, as was proposed in Ref. 34.

The ADAM optimizer was used with a learning rate of $10^{-4}$, $\beta_{1}=0.5$, and $\beta_{2}=0.9$. Common loss functions used for neural network applications to CT image data, such as for denoising, are explored in Ref. 36. To evaluate which loss function was most suitable for this task, four loss functions were evaluated after 100 epochs: MSE loss, L1 loss, VGG16 loss with feature extraction at the ninth layer, and a weighted combination of VGG16 and L1 loss, hereafter referred to as VGG16_L1 loss. Because VGG16 is trained with RGB images, the SPR map was repeated to three channels and normalized to match the statistics of the VGG16 training data, before being given as input to VGG16. The final network was trained with the VGG16_L1 loss, defined as

$$\mathrm{VGG}16\_L1=\lambda_{1}\mathrm{MSE}(\mathrm{VGG}16({\mathrm{SPR}}_{\mathrm{est}}),\mathrm{VGG}16({\mathrm{SPR}}_{\text{truth}}))+\lambda_{2}L1({\mathrm{SPR}}_{\mathrm{est}},{\mathrm{SPR}}_{\text{truth}}),$$

where MSE is the mean square error loss, L1 is the mean absolute error loss,^36^ ${\mathrm{SPR}}_{\mathrm{est}}$ is the estimated SPR map, and ${\mathrm{SPR}}_{\text{truth}}$ is the ground truth SPR map. $\lambda_{1}$ and $\lambda_{2}$ were both set to 1. Channel multiplication followed the scheme (1, 1, 2, 2, 2, 2, 2) for the resolution levels in the U-Net, with the number of initial features set to 64. The batch size was set to 2, and when training the final network, the number of epochs was set to 350.

We used two input channels because VMIs at two distinct energies were provided as input, and a single output channel because the corresponding ground truth SPR map is a single grayscale image. The original images were of 1024×1024 resolution, which required more GPU memory than what was available. Images were therefore re-sized to a resolution of 256×256. The network was subsequently trained on a Razer Blade 14, with an AMD Ryzen 9 5900 HX (3.30 GHz) processor with an NVIDIA GeForce RTX 3080 graphics card, and the training took ∼12  h.

Because of the stochastic gradient descent with random draws from the examples, as well as random initializations of the network parameters, every training instance results in a slightly different model. To get a sense of this uncertainty, the training was repeated fifteen times with identical data sets, settings, and noise realizations.

The XCAT phantom was transformed into a synthetic 70 keV VMI, using the theoretical attenuation coefficients computed from each material’s elemental composition. The attenuation map was then transformed into a synthetic VMI at 70 keV using the relationship

$$\mathrm{HU}=1000*\frac{\mu -\mu_{w}}{\mu_{w}-\mu_{a}},$$ (7)

where μ denotes the linear attenuation coefficient of the material, $\mu_{w}$ the linear attenuation coefficient of water, and $\mu_{a}$ the linear attenuation coefficient of air.

During the simulation process, the phantom is rescaled in relation to the original. To ensure pixel-to-pixel correspondence between the simulated CT image and SPR map prior to training, image registration was performed in MATLAB. In MATLAB, the built-in functions “imregconfig” and “imregtform” were used to generate a registration transform, fitting the synthetic VMI to the corresponding simulated VMI. The resulting registration transform was then applied directly to the SPR maps because these were of identical scale as the synthetic VMI generated from the XCAT phantom.

The data set was randomized and divided into a training and test set with a ratio of 80 to 20, using the built-in function “train_test_split()” from scikit-learn in Python. The training set was then divided into a training and validation set with the same ratio.

### 2.5 Non-Deep Learning Methods

To compare the accuracy of the network to that of conventional methods, a stoichiometric calibration and a VMI-based method originally adapted for DECT (hereafter referred to as N&A) were performed on the simulated PCCT data. A detailed description of these implementations is provided in the Appendix.

## 3 Results

### 3.1 Simulations

**Fig. 2:**
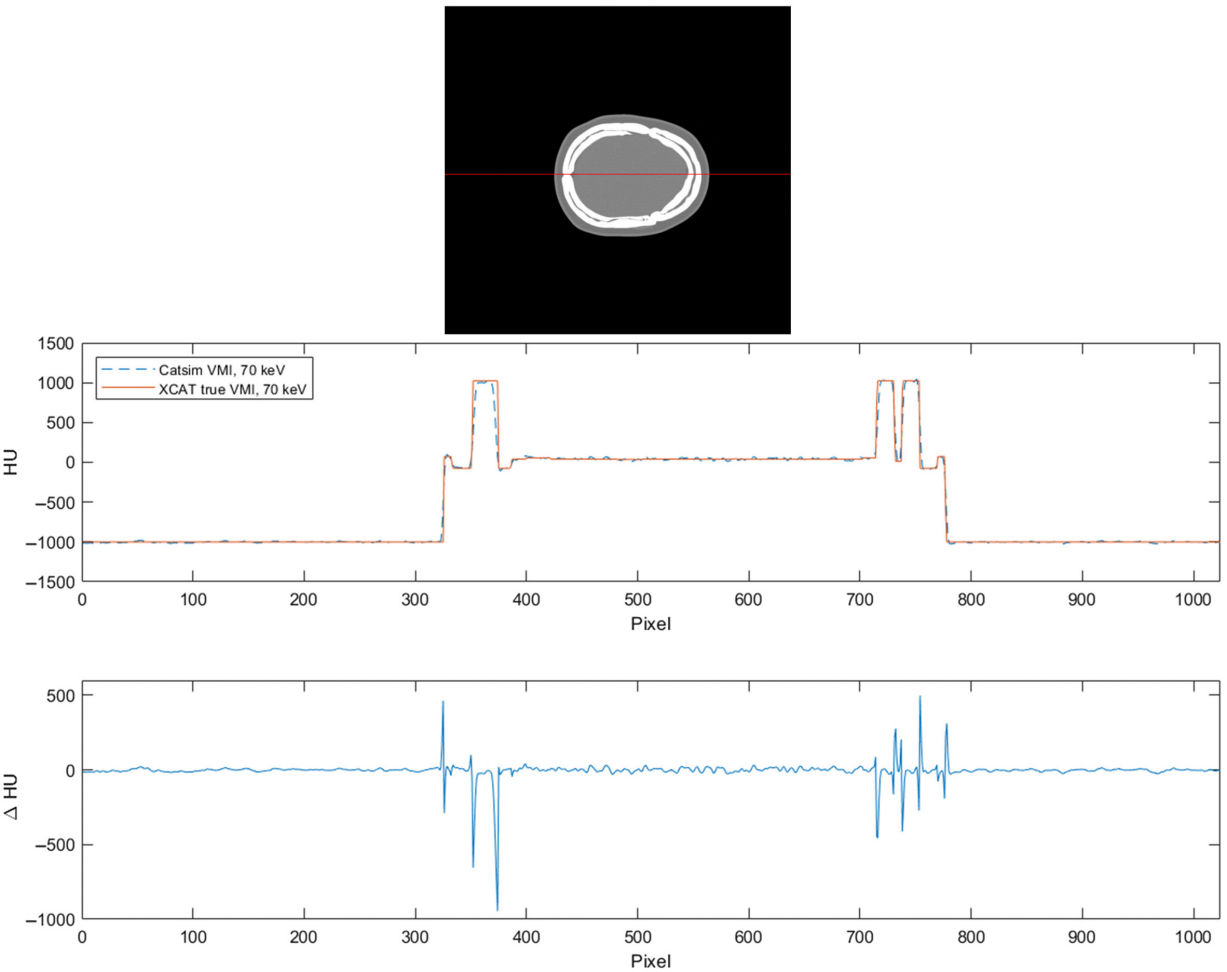
Plotted HUs for a true 70 keV VMI based on the XCAT phantom and a simulated 70 keV VMI from CatSim (WL: 0, WW: 2000).

To evaluate the accuracy of the photon-counting simulation, a synthetic VMI at 70 keV was computed from an XCAT phantom based on the material in each voxel. The HUs of a row through the image were plotted with the HUs of the corresponding row and slice from the simulated 70 keV VMIs. The results are shown in Fig. 2.

The RMSE of $\frac{\mu }{\mu_{\text{water}}}$ was calculated for each ROI, with μ calculated from the HU in the simulated VMI by inverting Eq. (7). The results are shown in Table 2.

**Table 2:** RMSE of $\frac{\mu }{\mu_{\text{water}}}$ calculated for each material’s ROIs.

Material	ROI 1 (%)	ROI 2 (%)	ROI 3 (%)
White matter	1.06	1.28	1.25
Gray matter	1.42	1.24	1.01
Brain (whole)	1.62	1.42	1.29
Skull	1.49	1.35	2.00

### 3.2 Performance of the Neural Network

The four different loss functions were each used to train the network and were subsequently evaluated. The mean RMSE for three regions of interest was calculated for the whole brain material for each loss function. The results are shown in Table 3.

**Table 3:** RMSE for the whole brain material for each of the loss functions.

Loss function	MSE	L1	VGG16	VGG16_L1
RMSE	1.30%	0.62%	0.24%	0.22%

**Fig. 3:**
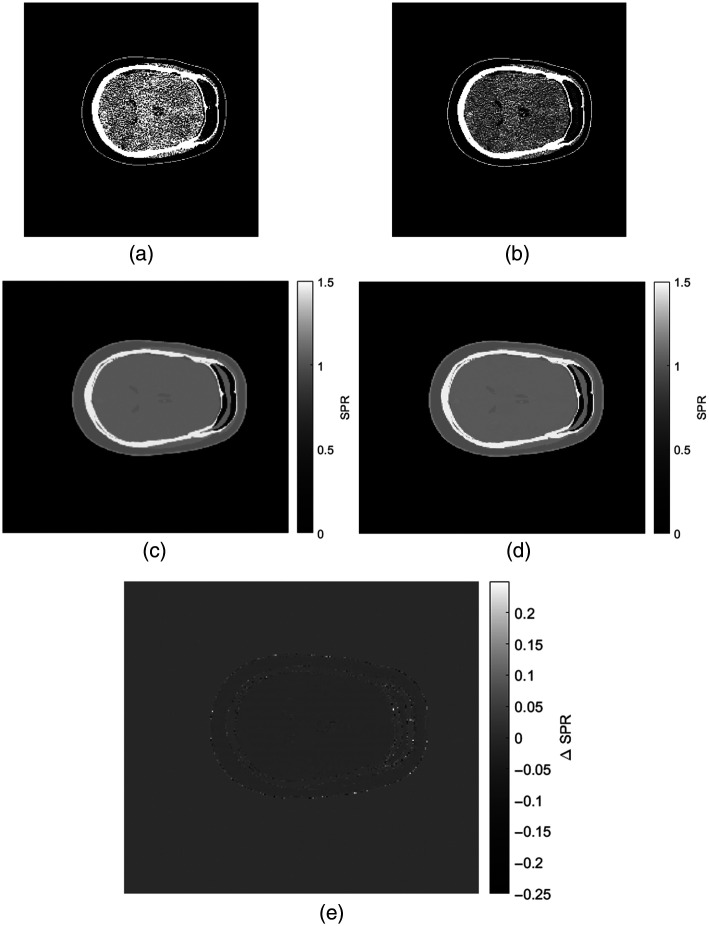
Observed 40 keV VMI (a) and 70 keV VMI (b), predicted (c), and true (d) SPR maps and a difference image (e) where true SPR values have been subtracted from the predicted values.

For further evaluation, the results of the best-performing loss function VGG16_L1 were used. Results for observed VMI, predicted SPR, true SPR, and their difference are shown for one selected slice in Fig. 3.

**Fig. 4:**
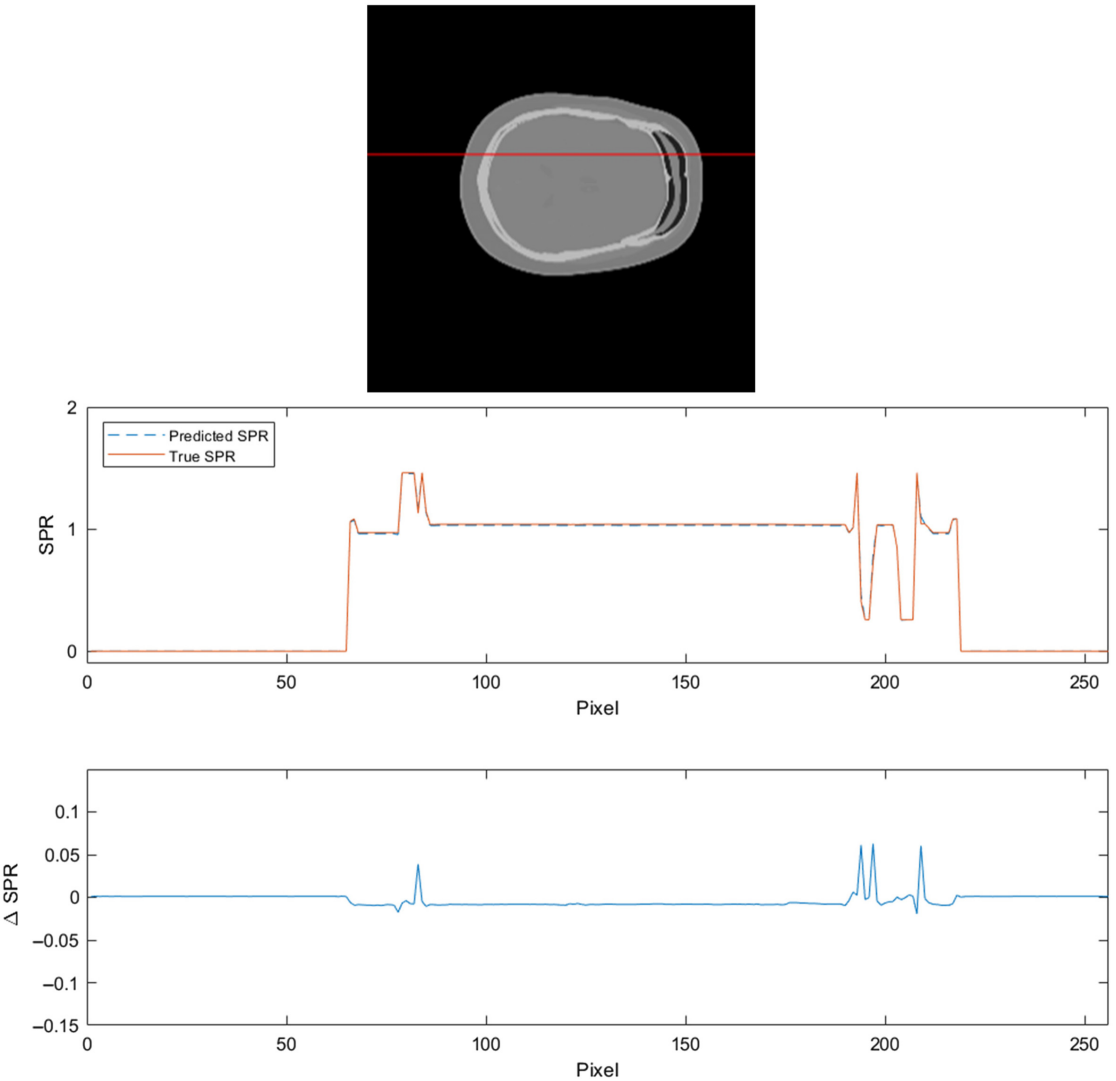
Comparison of predicted and true SPR values.

To evaluate the SPR prediction, a slice and a row were selected and the predicted and true SPR values were plotted. The result is shown in Fig. 4.

**Fig. 5:**
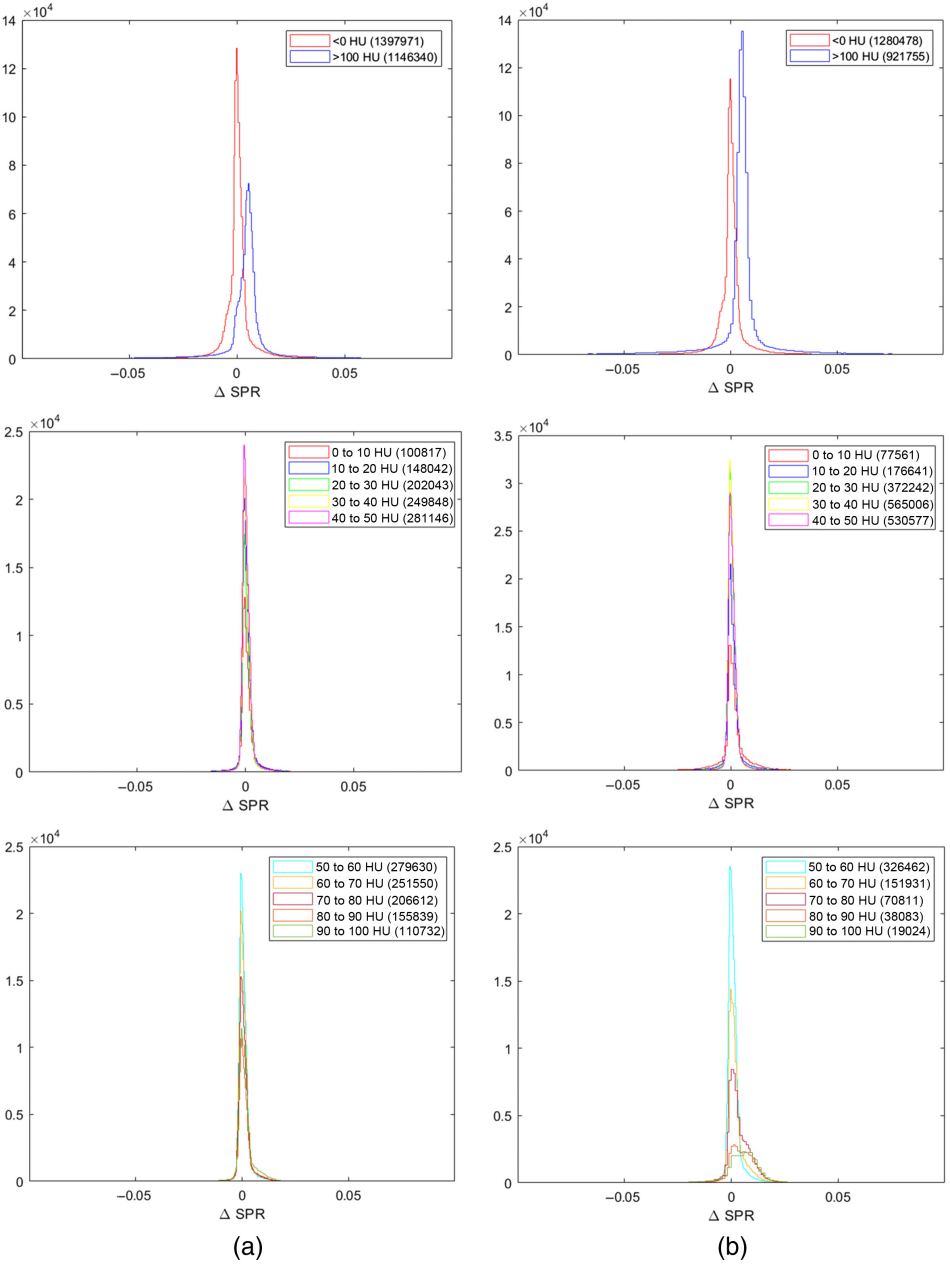
SPR error distributions for different HU intervals from the 40 keV (a) and 70 keV (b) VMIs. The parentheses denote the number of data points in the given interval. The maximal error was 0.59 and the minimal error −0.68.

Errors in SPR were calculated for tissues of lower density (HU below 0), higher density (HU above 100), and tissues in the range of 0 to 100 HU. Because most tissues fell into the 0 to 100 HU range, these were grouped into intervals of 10 HU for better visualization. Histograms of the error distributions are shown in Fig. 5.

The slice from Figs. 3 and 4 was evaluated over fifteen separate training sessions. Errors in SPR were calculated for white matter, gray matter, the whole brain material, and the skull, in three ROIs each. The RMSEs are shown in Table 4. For the relative error calculation, the mean predicted SPR in the ROI was taken as the predicted value. The results are shown in Table 5. The relative standard deviations, referring to a sample standard deviation for the ROI divided by the true SPR value, were calculated for each ROI and are shown in Table 6.

**Table 4:** Mean RMSE and standard deviation for ROIs of the white matter, gray matter, whole brain, and skull materials, in the SPR map predicted by the neural network. The column “mean” refers to the mean RMSE calculated over the three ROIs for one material.

Material	ROI 1 (%)	ROI 2 (%)	ROI 3 (%)	Mean (%)
White matter	0.26 ± 0.26	0.26 ± 0.27	0.27 ± 0.26	0.27 ± 0.26
Gray matter	0.26 ± 0.23	0.28 ± 0.26	0.27 ± 0.25	0.27 ± 0.25
Brain (whole)	0.27 ± 0.26	0.26 ± 0.25	0.30 ± 0.24	0.28 ± 0.25
Skull	0.36 ± 0.19	0.40 ± 0.24	0.41 ± 0.20	0.39 ± 0.20

**Table 5:** Mean relative errors and standard deviations for ROIs of the white matter, gray matter, whole brain, and skull materials, in the SPR map predicted by the neural network. The column “mean” refers to the mean relative error calculated over the three ROIs for one material.

Material	ROI 1 (%)	ROI 2 (%)	ROI 3 (%)	Mean (%)
White matter	0.25 ± 0.26	0.25 ± 0.26	0.26 ± 0.26	0.25 ± 0.25
Gray matter	0.25 ± 0.23	0.26 ± 0.26	0.25 ± 0.25	0.25 ± 0.24
Brain (whole)	0.26 ± 0.26	0.24 ± 0.25	0.24 ± 0.25	0.25 ± 0.25
Skull	0.23 ± 0.14	0.26 ± 0.17	0.25 ± 0.15	0.25 ± 0.15

**Table 6:** Mean and sample standard deviation of the relative standard deviations for ROIs of the white matter, gray matter, whole brain, and skull materials, in the SPR map predicted by the neural network. The column “mean” refers to the mean relative standard deviation calculated over the three ROIs for one material.

Material	ROI 1 (%)	ROI 2 (%)	ROI 3 (%)	Mean (%)
White matter	0.03 ± 0.01	0.02 ± 0.01	0.02 ± 0.01	0.02 ± <0.01
Gray matter	0.02 ± 0.01	0.04 ± 0.02	0.04 ± 0.01	0.04 ± 0.01
Brain (whole)	0.02 ± 0.01	0.02 ± < 0.01	0.12 ± 0.03	0.06 ± 0.01
Skull	0.07 ± 0.03	0.06 ± 0.01	0.11 ± 0.03	0.08 ± 0.02

Errors for the SPR maps estimated from the stoichiometric calibration and the N&A method were evaluated for the same slice and ROIs. The RMSEs are shown in Table 7, relative errors in Table 8, and relative standard deviations in Table 9. The optimal VMI pairs for the N&A method were 46 and 55 keV for the soft tissue and 51 and 61 keV for the bone.

**Table 7:** RMSE for ROIs using SPR calculation with the N&A method and SECT stoichiometric calibration.

	Material	ROI 1 (%)	ROI 2 (%)	ROI 3 (%)	Mean (%)
N&A	White matter	0.41	0.54	0.44	0.46
	Gray matter	0.71	0.62	0.58	0.63
	Whole brain	0.51	0.72	0.79	0.68
	Skull	2.78	1.64	3.00	2.48
Stoichiometric calibration	White matter	0.97	1.00	0.80	0.92
	Gray matter	1.17	1.04	0.83	1.01
	Whole brain	1.05	1.13	1.30	1.16
	Skull	0.40	0.64	1.22	0.75

**Table 8:** Relative errors for ROIs using SPR calculation with the N&A method and SECT stoichiometric calibration.

	Material	ROI 1 (%)	ROI 2 (%)	ROI 3 (%)	Mean (%)
N&A	White matter	0.21	0.41	0.09	0.23
	Gray matter	0.32	0.47	0.41	0.40
	Whole brain	0.01	0.51	0.02	0.18
	Skull	1.76	1.09	1.92	1.59
Stoichiometric calibration	White matter	0.02	0.18	0.24	0.15
	Gray matter	0.67	0.40	0.21	0.43
	Whole brain	0.09	0.25	0.16	0.17
	Skull	0.02	0.22	0.04	0.10

**Table 9:** Relative standard deviations for ROIs using SPR calculation with the N&A method and SECT stoichiometric calibration.

	Material	ROI 1 (%)	ROI 2 (%)	ROI 3 (%)	Mean (%)
N&A	White matter	0.34	0.33	0.42	0.37
	Gray matter	0.61	0.38	0.38	0.46
	Whole brain	0.51	0.48	0.78	0.59
	Skull	0.74	0.30	0.75	0.60
Stoichiometric calibration	White matter	0.96	0.97	0.74	0.89
	Gray matter	0.93	0.93	0.79	0.88
	Whole brain	1.03	1.08	1.27	1.13
	Skull	0.28	0.39	0.85	0.50

## 4 Discussion

The network estimated SPR with mean RMSEs of 0.26% to 0.30% for the soft tissue and 0.36% to 0.41% for the bone, as seen in Table 4. Moreover, the proposed method was quite robust to image noise, yielding high accuracy estimates despite the inclusion of realistic noise levels for PCCT in the simulations. Comparing the RMSEs in Tables 2 and 4, it is clear that the network did not amplify noise and bias from the VMIs.

In Fig. 4, it can be seen that SPR errors are generally very low, with peaks close to high-density gradients. In these regions, the SPR tends to be overestimated, which would amount to accumulated error rather than being averaged when integrating over a proton beam path. On the one hand, if aiming to be an applicable method for the head, where the soft tissue and cavities are enveloped in the dense bone, the ability to handle steep density gradients would be a prerequisite. On the other hand, for the VMI in Fig. 2, there are high HU errors close to the high-density gradients as well. Some level of SPR error in these regions might be difficult to mitigate if it originates from the CT image data. However, for the reasons above, it would be preferable if errors were random rather than displaying a general overshoot.

In Table 4, it can be seen that the standard deviations were similar to the means, showing quite a large spread in performance over the 15 training sessions. Although more effort should be devoted to developing an optimal model for this task, the lack of precision in the training may not be an issue in a clinical setting where it is possible to select the best-performing model.

When comparing the network performance to that of stoichiometric calibration and the N&A method, the network’s SPR estimates had generally lower errors, as seen in Tables 4 and 7, and less noisy predictions, as seen in Tables 6 and 9. This can likely be attributed to the network’s ability to learn from not only the quantitative information but also the geometry and high-level features of the CT images. This likely enables some mitigation of noise and partial volume effects in the CT image data that are otherwise propagated to the SPR map through the pixel-wise conversion of HU. Although a major advantage of the deep learning approach, there may be a precariously fine line between valuable information complement and undesirable information synthesis (e.g., if a network model is implemented on a novel patient geometry not previously encountered during training and makes inapplicable assumptions about high-level features), which emphasizes the need for careful assessment of domain validity before implementing these models.

As seen in Tables 7 and 8, the HLUT implementation by Peters et al.^37^ with the simulated PCCT VMI demonstrated very good performance, especially for the bone tissue. This raises the question of whether the best HU-SPR conversion method is a multi-variable problem formulation that makes explicit use of spectral information, or if a well-formulated SECT-based method is a successful approach as long as the input CT data is of high quality (as a result of the quantitative information). It should be noted, however, that by using simulated CT acquisitions of digital phantoms, the usual vendor-specified uncertainty in density and/or composition of the calibration inserts has been eliminated. The three-parameter model may be over-fitted to the calibration data points, which likely enabled a very exact model in this case due to idealizations in phantom data and simulations.

The N&A method performed well for soft tissues when used with simulated PCCT VMI, while the results for the bone were less accurate than previously reported. Worth noting is that the RMSE in this work was calculated over an ROI in the SPR map, i.e., no averaging was made in either CT image input or final SPR map, while Näsmark and Andersson estimated RMSE from ROIs in different phantom inserts of the same type (lung, soft tissue, and bone, respectively). The RMSE in this work thus includes both noise propagated from CT image data as well as bias error from the method. With generally noisier HU for high-density bone compared with the soft tissue, this likely contributed to the large discrepancy in reported RMSE for the bone. We also noted a dependency on the inserts included in the optimizations, where the inclusion of only the least dense bone insert yielded an optimal pair of 123 and 127 keV (which ranked 84 in the original optimization), yielding RMSEs for the XCAT phantom of around 1%. In conclusion, it seems that the optimization process in its current form may be sensitive to the choice of optimization inserts, where the VMI pair ranking is not necessarily valid for the generalized case (or for the RMSE calculation in this work).

Estimating the clinical implications of the achieved accuracy is not straightforward as most work on the topic applies a combined uncertainty budget while this work has calculated errors for the SPR estimates only. In Ref. 38, the authors compared the robust optimization of proton treatment plans with 3% and 2% range uncertainty, the lower uncertainty enabled by a vendor’s built-in SPR algorithm for DECT. A clinically relevant dose reduction was achieved in one or more organs at risk for 89% of patients when reducing the uncertainty, with an expected decreased toxicity level for 44% of the patients. In Ref. 39, the authors found that the N&A method outperformed the accuracy achieved by the vendor’s algorithm. In this work, we compared the N&A method with the neural network using the same data and found that the network achieved higher accuracy. We therefore expect a neural network to achieve at least comparable clinical benefit as the vendor’s algorithm, with potential for further improvement.

As for the usage of simulations and digital phantoms in general, an important limitation to consider in the present work is the idealizations of simulations and training data. The proposed method solves the ground truth problem, but may simultaneously introduce issues of transferability to the clinical situation. Furthermore, for this proof-of-concept work, the effects of pileup and crosstalk were excluded, which is an important idealization of the data possibly impacting the results. This was in part motivated by these effects having a low impact on the final reconstructed images as the system is able to correct for artifacts quite well, as discussed in the Appendix. For the end goal of clinical use, however, it is crucial to ensure high-fidelity simulations that accurately incorporate all the phenomena that may impact the image quality and quantitative information, as well as evaluation on physically scanned rather than simulated image material.

Furthermore, the tissue compositions used in the study are listed standard compositions, based on the non-pathological case. The training data do not take into account either realistic patient-to-patient tissue variation or the potential effects of cancer. Tumors may also introduce variation in the patient geometry, with possible impact on the results which should not be overlooked. The lack of representative complexity in the data set is a limitation of this study and will be addressed in future work. However, given the SPR error distributions for different HU intervals shown in Fig. 5, we expect low errors also for the tumor tissue which is expected to lie within the range of HU included in this work. Important to note is also that the patient variations used for the phantoms were all adult—no children or other age groups were included in the data set. Considering the potential benefits of proton therapy and minimal radiation exposure for pediatric patients, additional effort should be devoted to an adaptation of the model to these patient geometries.

Even though the results of this simulation study are not directly comparable to errors reported for experimental studies with DECT (0.7% to 1.5%), the results have shown the potential of further improving SPR estimate accuracy using deep learning. Experimental evaluation of the network with real scanned material will be left to future work.

## 5 Conclusion

In this proof-of-concept work, the proposed method, which used simulated PCCT images and deep learning for SPR estimation, was shown to improve on results for conventional methods developed for SECT and DECT performed on the simulated PCCT data. Although there are idealizations in the simulations and training data that call for further elaboration, these early results demonstrate the potential for a combination of PCCT and deep learning for improving the accuracy of SPR estimation and thereby reducing the beam range uncertainty in proton therapy treatment planning.

## 6 Appendix

### 6.1 Stoichiometric Calibration

It has been reported that a lack of consensus regarding the implementation of stoichiometric calibration has led to treatment center inter-variability.^3^ To avoid ambiguity, the standardized guide by Peters et al. in Ref. 37 was closely followed, using the available code and Excel input template on GitHub (https://github.com/CTinRT/HLUT-guide). A Gammex Advanced Electron Density phantom was defined in CatSim, with insert material compositions in accordance with those listed in the input template on GitHub. The elemental ionization energies were adjusted manually to correspond to the values used for the other SPR calculations in this work.

PCCT acquisitions to specify the HU for lung, soft tissue, and bone inserts were simulated in CatSim for several insert configurations, as suggested in Ref. 37. The CT protocol parameters were the same as for the XCAT CT acquisition. Twelve slices were acquired for each configuration. Reconstructed basis images for PE and PVC were then recombined into 70 keV VMIs, and the HU for a tissue insert was calculated as the mean value of a circular ROI covering ca. 70% of the insert’s inner cross-sectional area, averaged over the 12 slices.

The stoichiometric calibration was performed with the code available on GitHub. The only changes made to the script were the Mayneord factor, used for the effective atomic number calculation, which was set to 3.21 (as in Ref. 33), and β which was set manually to the same value as for the other methods in this work. The HLUTs were then calculated separately for the head and body phantom. Because this work only included XCAT phantoms of the head, the head HLUT was selected for further evaluation. Based on the given breakpoints of the line segments, a piecewise linear function was defined using the “pwlf” package in Python. The resulting function was subsequently used to predict SPR maps based on simulated 70 keV VMIs of the XCAT phantom.

### 6.2 SPR Estimation from Pairs of VMIs

A VMI-based method originally adapted for DECT was applied to the simulated PCCT image data. The proposed method by Näsmark and Andersson, described in full detail in Ref. 33, employs pairs of VMIs to calculate the effective atomic number and relative electron density. The mean ionization energy is derived from the effective atomic number via separate parameterizations for soft and bone tissues, after which the mean ionization energy and relative electron density are used as input quantities to Eq. (3).

In the same work, the authors demonstrated that the SPR accuracy was highly dependent on the VMI pair, and the optimal choice depends on the scanner’s ability to generate accurate VMIs for different energies. An initial optimization was performed on VMI pairs in the range of 40 to 140 keV, where each pair was assessed based on its accuracy in estimating the SPR of the tissue inserts. The optimal pair for each tissue category (lung tissue, soft tissue, and bone) was selected as the one yielding the smallest RMSE, as compared with the ground truth calculated with Eq. (3) based on the elemental composition of the inserts and listed reference tissues from Ref. 26. We did not have to explicitly enforce the skewness criterion recommended in Ref. 39 because this was fulfilled by the highest ranking VMI pair anyway. The same Gammex phantom image data obtained for the stoichiometric calibration was used for the optimization. Because this work included only XCAT head phantoms, the optimization was based on the Gammex head phantom acquisitions.

### 6.3 Effects of Crosstalk and Pileup

**Fig. 6:**
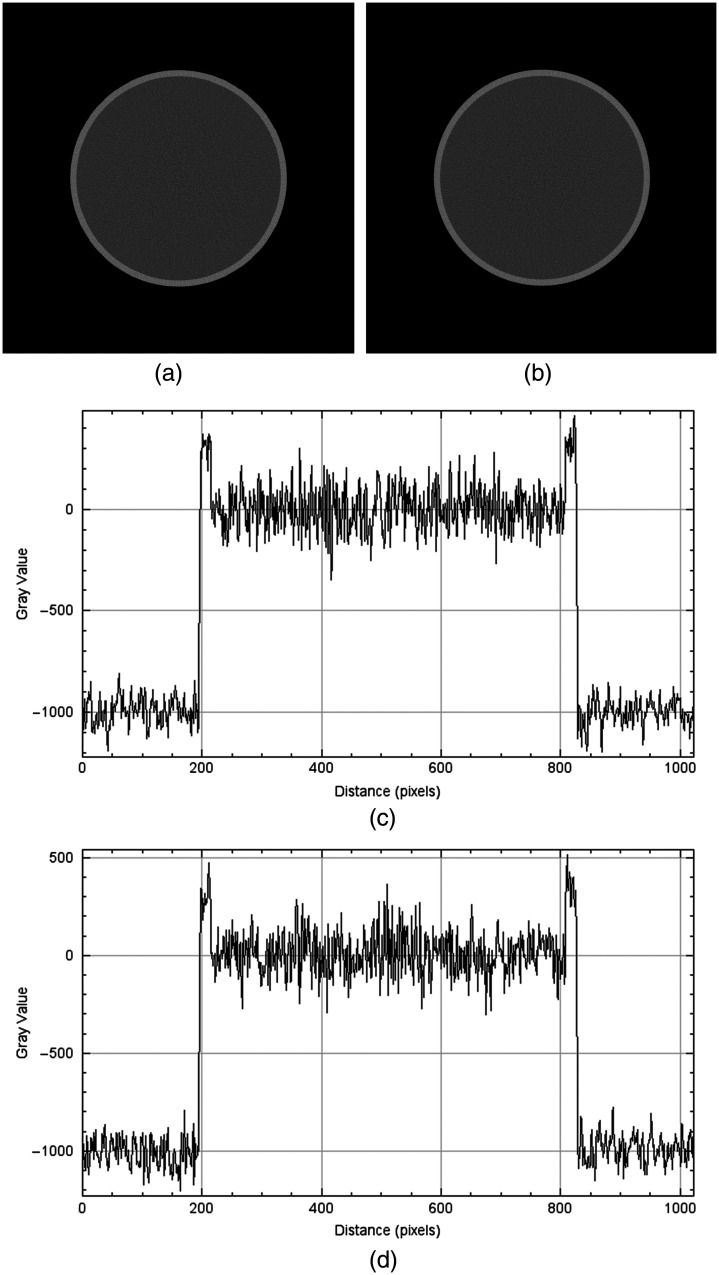
Reconstructed VMIs at 70 keV for a simulation excluding (a) and including (b) crosstalk and pileup, as well as line profiles through the widest part of the cylinder for the simulation excluding (c) and including (d) crosstalk and pileup.

We chose to exclude crosstalk and pileup in the simulations, which are known to deteriorate the image quality in PCCT. This choice was motivated by the fact that the effects are negligible in the final reconstructed image, after the application of correction algorithms in the image chain. We simulated a scan of a water phantom to validate this assumption, by first excluding crosstalk and pileup, and then including these effects. A pileup correction algorithm was applied in the reconstruction.^40^ The example is shown in Fig. 6.

## Data Availability

Code and the network’s estimated SPR maps are available at https://github.com/KTH-Physics-of-Medical-Imaging/SPR.
